# Conceptual Framework to Support Clinical Trial Optimization and End-to-End Enrollment Workflow

**DOI:** 10.1200/CCI.19.00033

**Published:** 2019-06-21

**Authors:** Neha M. Jain, Alison Culley, Teresa Knoop, Christine Micheel, Travis Osterman, Mia Levy

**Affiliations:** ^1^ Vanderbilt University Medical Center, Nashville, TN; ^2^ Rush University Medical Center, Chicago, IL

## Abstract

In this work, we present a conceptual framework to support clinical trial optimization and enrollment workflows and review the current state, limitations, and future trends in this space. This framework includes knowledge representation of clinical trials, clinical trial optimization, clinical trial design, enrollment workflows for prospective clinical trial matching, waitlist management, and, finally, evaluation strategies for assessing improvement.

## INTRODUCTION

Approximately 40% of cancer-related clinical trials terminate prematurely,^1^ which results in an immense loss of time, money, and human effort. Failure to optimize clinical trial design, inefficient enrollment processes, and poor accrual rates are the main reasons for premature trial closure.^1^ As successful trial accrual is paramount to drug discovery, efforts to address this challenge are urgently needed. Informatics tools can improve accrual and facilitate workflows to dramatically enhance the outcome of the clinical trial enrollment process. Without automated systems for supporting the entire breadth of the clinical trial enrollment workflow, it is only possible to make incremental improvements to the efficiency of the accrual process. In this work, we present a conceptual framework to support clinical trial optimization and end-to-end enrollment workflows. We review the current state, limitations, and future trends in methods to support clinical trial optimization and enrollment. As one of the early adopters of next-generation sequencing (NGS) testing and biomarker-driven clinical trial matching, we at the Vanderbilt-Ingram Cancer Center are in a strategic place to share insights and improvements from our own trial-matching experience.

## CONCEPTUAL FRAMEWORK

**FIG 1.**
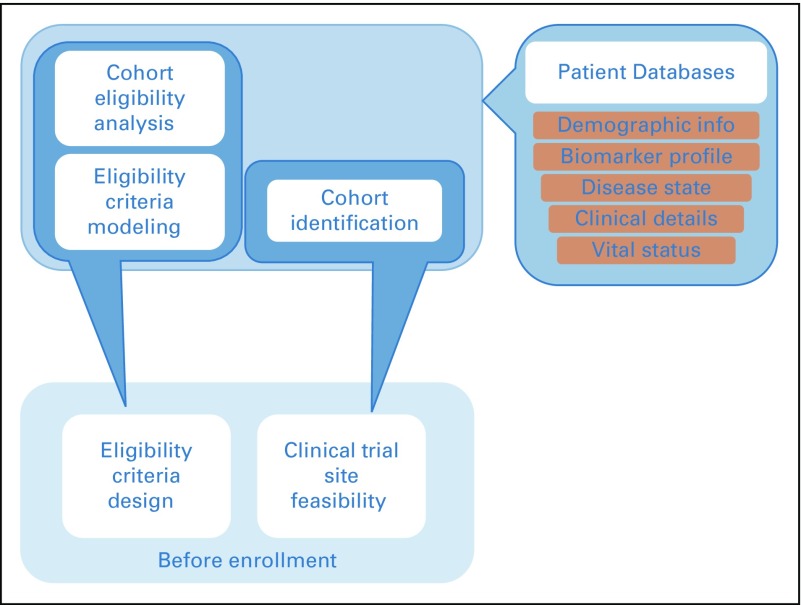
Clinical trial optimization. Key components for eligibility criteria, design optimization, and evaluation of clinical trial site feasibility.

Before opening for enrollment, a trial should undergo rigorous design optimization and assessment of site feasibility. Eligibility criteria design optimization can be defined as optimizing the study to maximize enrollment, improve efficiency, and mitigate risks of premature closure by using data analytics, artificial intelligence, or other big data solutions. Site feasibility assessment refers to the decision to open a trial at a given institution after careful consideration of the existing portfolio of potentially competing trials, institutional strengths, and whether there is an unmet but well-recognized clinical need (Fig 1).

**FIG 2.**
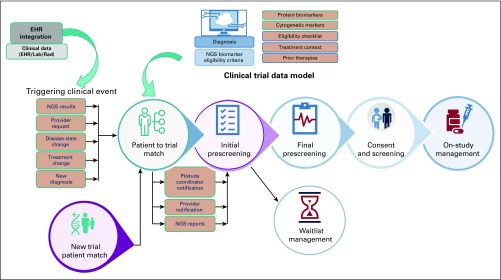
Overview of the trial enrollment process. Trial enrollment begins with a triggering clinical event—next-generation sequencing (NGS) test result, request from provider, change in disease state or treatment, a new diagnosis, etc—or opening of a new clinical trial at an institution. This query results in a list of potentially eligible trials on the basis of clinical/genomic data from the patient as well as specific eligibility criteria for individual trials. Patient-specific information is obtained from the electronic health record (EHR) and may be in the form of a laboratory report (Lab), a radiology report (Rad), or a clinical note. Clinical trial information is pulled from a structured clinical trial data model. Once a list of trials is identified, clinical research personnel narrow this list by performing multiple rounds of prescreening and screening to evaluate patient eligibility while maintaining communication with the patient’s provider and study coordinator to ensure the availability of slots in clinical trials and continued patient eligibility. At this point, a patient could be waitlisted if the identified trial is a future line of treatment or the trial itself is suspended.

End-to-end clinical trial matching workflow is complex and consists of several components (Fig 2). The process typically begins when a provider tries to find a clinical trial for his or her patient. Other events can also set this process in motion, such as new diagnoses; a change in disease state; new test results, such as an NGS test; or a new trial opening. Whatever the triggering event, this initial query results in a list of potential trials on the basis of the patient’s clinical or genomic data. As trial eligibility criteria have become increasingly stringent, a significant amount of human effort is required at this step to refine the trial list and generate meaningful trial recommendations for patients. Once such a trial is identified, results are shared with the patient’s provider, who then makes an informed decision about whether to move forward with the trial and, if so, requests a detailed prescreening for the patient. Alternatively, the provider may choose to consider the trial as a future line of therapy or deem it inappropriate. After the successful completion of detailed prescreening, the patient is contacted and offered the option to enroll. Interested patients undergo a final screening for evaluation of modifiable health criteria followed by a consenting process. Successful completion leads to patient enrollment in the trial. Wait-list options are frequently used in this workflow—namely, when trials have limited slots or when the provider decides to pursue a trial opportunity as a future line of therapy for the patient.

To improve precision and recall for the clinical trial matching process, a robust clinical trial data model (CTM) and an exhaustive patient data model are required. The CTM stores accurate and updated information about clinical trial eligibility and recruiting statuses and is therefore a crucial part of the enrollment workflow. A highly structured CTM allows users to refine trial results in an automated manner, thereby significantly reducing the human burden of initial prescreening and shortening the timeline for the enrollment event.

Clinical trial enrollment is an assiduous process that involves having up-to-date knowledge of the patient’s disease state for accurate trial prediction, multiple rounds of eligibility screening, and multiple communications between the patient, the patient’s provider, the trial investigator, and clinical research staff. On average, clinical research personnel spend 3.4 to 9 hours screening and enrolling a patient in a clinical trial.^2^ The enrollment process is rendered even more complex by the need to evaluate patients for multiple clinical trials, each of which has its own set of inclusion and exclusion criteria. Furthermore, as the disease state of a patient changes as a result of intervention or disease progression, his or her eligibility changes and the patient may be eligible for a completely different set of trials. A robust and meticulously curated patient data model would house clinical, genomic, and treatment-related data in a structured fashion.

As precision oncology moves from targeted panels to whole-exome sequencing and ultimately to whole-genome sequencing, eligibility criteria of trials and the screening efforts involved may become increasingly complex. A considerable amount of progress has been made in the development and use of informatics-based tools for refining trial options for patients—for example, My Cancer Genome, MatchMiner, IBM Watson, TrialProspector, and The Jackson Laboratory (JAX) Clinical Knowledgebase (CKB) database, among others.^3-6^ However, tools and systems to support aspects of the trial enrollment workflow beyond matching—namely, prescreening and screening processes, provider communication systems, and management of trial waitlists—are in their infancy.

## KNOWLEDGE REPRESENTATION OF CLINICAL TRIALS

Accurate knowledge representation can refine trial search results by improving precision and recall, thereby reducing the burden of manual prescreening and enhancing the possibility of trial enrollment.

### Clinical Trial Statuses

There are two aspects of information about clinical trials that must be collected and reported accurately for improving accrual: recruiting status–related information (eg, overall recruitment status, cohort status, slot availability, etc) and patient eligibility–related information (eg, clinical details, disease status, prior treatments, and other inclusionary/exclusionary criteria). A single clinical trial may have many enrollment sites. Although clinical trial recruitment status for each of these sites is currently publicly available through the National Library of Medicine (NLM)^7^ and the US National Cancer Institute (NCI) Clinical Trial Reporting Program,^8^ there can be a delay of approximately 5 months for recruitment updates from local sites to be reflected on these resources.^9^ This could result in inaccurate trial matches and misdirected enrollment efforts. Furthermore, for studies with multiple cohorts or arms, such as early-phase studies and multidisease trials, there is an additional burden of tracking slot availability and recruiting status of individual cohorts/arms. A consolidated version of this information for all participating sites is not currently available from any commercial or publicly available resource.

In our own trial-matching experience, 46% of false-positive trial matches could be attributed to the lack of updated information on cohort-level trial recruiting statuses (unpublished data). Using automated systems to track cohort-level eligibility statuses across various study sites could vastly improve the precision of trial-matching events.

### Clinical Trial Documents

Publicly available clinical trial eligibility documents are inherently unstructured and nonstandardized—inexplicable abbreviations, lesser-known gene aliases, and ambiguous language are common. The word limit imposed by the ClinicalTrials.gov Web site and frequent addendums to protocol documents add to the complexity. There have been several approaches to standardize the information in clinical trial documents. Clinical trial markup language, developed in association with MatchMiner, enables the structured coding of clinical trial information.^3^ The Trial builder module from Trial Prospector contains a library of 32 reusable Boolean or integer criteria^5^ to facilitate trial annotation. IBM Watson and TriNetX have used natural language processing (NLP) and semantic-based technology, respectively, to convert unstructured documents into structured annotations (Table 1). My Cancer Genome, in collaboration with GenomOncology,^10^ has developed a semiautomated approach in which entity tagging is used to refine trial results before they are manually curated into structured annotations.

**TABLE 1.**
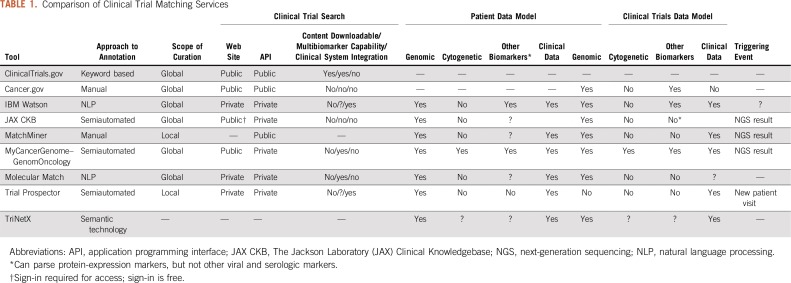
Comparison of Clinical Trial Matching Services

Development of protocol-authoring tools that allow users to create reusable eligibility criteria sets that are capable of handling Boolean, integer, date, and other relevant data types is ultimately needed to make machine learning more effective. An ideal protocol builder would enable users to catalog protocol addendums and track different versions of trial documents. Adoption of such tools at the level of the NCI and NLM and their subsequent dissemination for downstream use by individual institutions could greatly benefit the oncology community.

## CLINICAL TRIAL OPTIMIZATION AND TRIAL DESIGN

Rigorous clinical trial design optimization before trial opening is crucial to the success of trial enrollment. Newly opened trials should be in alignment with institutional needs, which are ultimately defined by the characteristics of the patient population. Recently, patient health records have been leveraged to gain valuable insight into an institution’s patient population.^11-14^ These large data sets provide valuable data and are powerful tools for discovery and cohort identification.

### Patient Databases

Patient databases are comprised of structured or unstructured data arranged in a hierarchical or relational model. Data could be deidentified or may contain patient identifiers, depending on the end use of the data set. A deidentified version could suffice for research purposes and for assessment of trial design and optimization, treatment trends, and more, whereas an identified database could be used for identifying patient cohorts and, subsequently, for enabling clinical decision support for patient enrollment in clinical trials. These databases could house an institution’s own clinical data, made available to researchers within the institution, or a larger, pooled data set from multiple institutions. Such databases could be centralized or decentralized. In a centralized database, such as the American Association for Cancer Research Genomics Evidence Neoplasia Information Exchange (AACR-GENIE),^15^ all users and sites send data to a central data repository. Conversely, in a decentralized database (eg, TriNetX^16^) data are shared between centralized networks. A decentralized network could either be a federated network (eg, Global Alliance for Genomics and Health^17^) or a distributed network (eg, International Cancer Genomics Consortium^18^). The AACR-GENIE data set is an international collaboration between 19 sites and approximately 59,437 clinical sequencing samples from patients with cancer and offers the opportunity to study rare mutations. In a simulated clinical trial matching experiment performed over the first 18,000 GENIE patients to the NCI-MATCH trial (ClinicalTrials.gov identifier: NCT02465060), we found that three of the 26 arms matched to fewer than five patients^19^. This illustrates how modulating the eligibility criteria design on the basis of the target population can prevent trials from being overly exclusionary.

To engineer a database most suited for assessment of clinical trial optimization and cohort identification, it is important to understand the design of the data set—in other words, the data model, data collection method, and data quality.

Patient data models contained in databases can house a variety of data (Table 2). An ideal patient data model would have the ability to be queried with fully structured data; however, in the real world there is an abundance of unstructured data in electronic health records (EHRs). Current strategies to circumvent this challenge have focused on the manual curation of data and the use of text-mining approaches, such as NLP, for data abstraction.^20-22^ IBM Watson uses NLP to populate structured data fields in a patient data model by mining unstructured notes from patients’ medical charts. The MyCancerGenome–GenomOncology collaboration has used a semiautomated approach in which diagnosis, genomic markers, and demographic data are populated by parsing structured versions of NGS test reports (ie, JavaScript Object Notation [JSON] and eXtensible Markup Language [XML]) while other components are manually added in a structured format after a detailed patient chart review. Although manual curation is the gold standard for data abstraction, it is expensive, subject to error, and time consuming. Rigorously tested NLP approaches can be both time efficient and relatively error free, but these approaches lack the refinement required for deciphering complex unstructured data. In the past decade, however, NLP approaches have reached a new level of sophistication and have been used widely for data abstraction.^11,13,14,21,23^ Employing the ensemble method of using NLP to extract and narrow down relevant data elements, then using human curation for review and refinement of results could be an efficient and feasible solution. Until such efforts as Minimal Common Oncology Data Elements (mCODE), which allow patient data extraction across EHRs, are fully implemented, we need alternative solutions to cope with this information gap.^24^

**TABLE 2.**
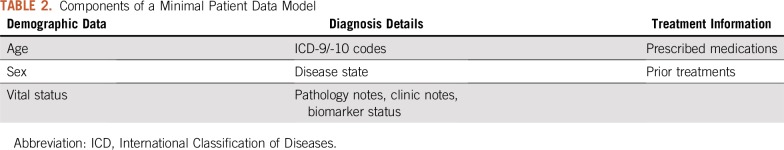
Components of a Minimal Patient Data Model

### High-Yield Data Elements for Minimal Patient Data Model

Data curation is labor intensive and expensive; therefore, identifying data elements that have the highest value for the intended downstream application is of high importance. Vital status, disease state, and biomarker status are high-yield data elements that can heavily influence predictions regarding trial enrollment.

#### Vital status.

Publicly available, free sources of vital status data, updated in real time, are needed urgently, as vital status in the EHR is frequently outdated. State registries often lag in reporting vital status because of infrequent updates. Estimates of vital status can be automatically extracted using the date of last follow up, date of last encounter, date of prescription refill, or other similar surrogate events. Informatics tools that use data from the National Death Index, Social Security Death Index, and online obituaries could be helpful in updating vital status. However, these data sets are restricted for use in statistical analysis and reporting, which makes them extremely limiting. In an internal prospective trial matching study, we discovered that 21% of patients prescreened for clinical trials were already deceased (unpublished data). Updated information about patient vital status can minimize such misdirected enrollment efforts.

#### Disease state.

**FIG 3.**
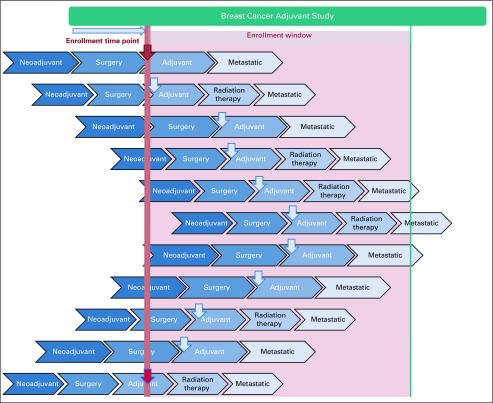
Concept of enrollment window. The red downward pointing arrow shows eligibility at a specific time point, as opposed to the blue arrows that show eligibility over a specific enrollment window (depicted by the shaded panel). The concept of enrollment window takes into consideration the temporal nature of a patient’s disease state.

Current approaches to clinical trial optimization and estimation of patient cohorts are focused on the patient population in a specific disease state at a discrete time point. This may result in inaccurate estimations as a result of the temporal nature of the disease state. We envision trial optimization having a time-related parameter and evaluate it is as the eligibility of a patient population for the trial of interest over a specified interval of time (Fig 3). As this enrollment window can directly affect trial optimization, it should be carefully assessed on the basis of accrual goals, open enrollment sites, and target patient population.

#### Biomarkers.

As NGS testing becomes the standard of care for diseases, such as lung cancer and melanoma, there is greater incentive to capture the biomarker profile of patients in a computable form. Biomarker status can play an influential role in accurate diagnosis, determining prognosis, informing treatment decisions, and predicting drug responses and treatment outcomes. Several patient data models (Table 1) have incorporated structured genomic biomarkers. We have extended our biomarker model to include protein, viral, serologic, and other cytogenetic markers, and have also chosen to curate at the variant level. Using variant-level biomarker status—for example, BRAF V600E—as opposed to gene-level biomarker status—for example, BRAF mutated—can be particularly helpful illuminating the real frequency of extremely rare mutations and in certain cases provide evidence to document their relative abundance. Access to large patient cohorts can accelerate this discovery process and pave the way for the wider application of targeted therapies.

As patient data are rarely complete, there is much interest in designing robust tools that are capable of handling missing data. We propose the concept of “partial match” to define events in which patients seem to qualify for a trial but have missing data fields that make it hard to draw definitive conclusions about eligibility. Instead of excluding these patients altogether, they should be considered partial matches. For example, a patient with breast cancer with unknown androgen receptor status should be classified as a partial match patient who needs additional testing to confirm eligibility for an androgen receptor–driven breast cancer trial. Furthermore, as bigger gene panels enter the clinic, it is important to distinguish between biomarkers that were tested but not detected versus biomarkers that were not tested at all. Elucidating this distinction can improve trial optimization and cohort identification efforts.

#### Treatment history.

A patient’s treatment history can significantly affect trial eligibility. Accurate and structured records of previous lines of therapy, drugs, duration of treatment, etc, can improve the precision of clinical trial matching tools.

### Cohort Identification and Cohort Analysis

Informatics tools that can query large data sets could be a valuable simulation tool for cohort identification, informing decisions about the number of sites required to be opened and the enrollment window needed to complete accrual.

Such tools as Record Counter,^25^ i2b2,^26^ and others allow researchers to query over aggregated, deidentified patient data to identify a patient cohort with specific clinical, demographic, or other molecular features. These queries apply logical combinations of constraints to the query—that is, AND, OR, NOT—and are equipped to add temporal constraints to the data being queried—for example, no administration of drug X in the last 6 months; creatinine level less than 2.0 mg/dL—as well as modulate a particular criterion and observe its impact on the eligible patient pool (eg, its impact on the size of the patient cohort for creatinine levels of 1.7 mg/dL *v*. 2.0 mg/dL). Defining a time interval for recruitment while identifying a cohort could provide a realistic estimate of the enrollment window, which could be unique on the basis of each participating site and its characteristic patient population.

### Modeling Eligibility Criteria for Clinical Trials

There have been two major approaches to modeling eligibility criteria: a rule/query-based approach and an assertion-based approach. A query-based approach uses NLP and informatics tools to find information from structured and unstructured data elements in the EHR, whereas an assertion-based approach involves creating structured elements on the basis of unstructured data, which can be tagged and queried in multiple ways. The computable format of the assertion-based approach allows data to be used for several downstream applications, optimizing both trial search and trial matching events. We have annotated 3,950 clinical trials in the MyCancerGenome knowledge base using an assertion-based approach.^6^

To improve treatment options for patients and accrual, NCI recently published recommendations for broadening eligibility criteria around brain metastases, HIV positivity, minimum enrollment age, prior malignancies, and organ dysfunction.^27,28^ The Targeted Agent and Profiling Utilization Registry Study (TAPUR) protocol by ASCO has minimal exclusions in these categories.^29^ Such inclusive trial designs not only benefit enrolled patients, but also create evidence for the generalizability of a drug for broader populations.

## ENROLLMENT WORKFLOWS

Once a clinical trial has been successfully evaluated for optimization and an appropriate enrollment window defined, patient enrollment efforts are initiated. The provider, trial investigator, trial sponsors, or patients can drive these enrollment workflows.

### Workflow Models for Prospective Clinical Trial Matching

**FIG 4.**
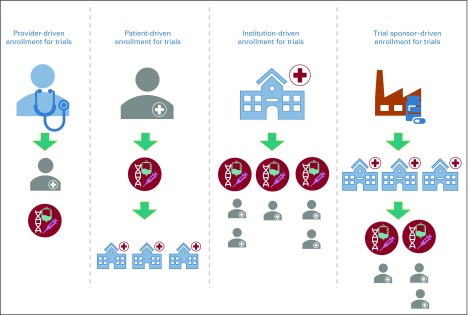
Workflow models for supporting clinical trial enrollment. Provider-driven enrollment occurs when providers find trials for their patients at their own institutions. Patient-driven enrollment occurs when patients seek out trials across many institutions. Institution-driven enrollment occurs when an institution seeks to enroll patients across its entire portfolio of open trials. Trial sponsor–directed enrollment occurs when trial sponsors work to identify sites that would result in successful completion of trials by recruiting the appropriate patient population. Clinical trials are indicated by red circles with the gene and syringe icon.

There are various workflow models that can support enrollment in a clinical trial (Fig 4). The more discernible approach is provider-driven enrollment for which the workflow is oriented toward finding a set of trials appropriate for a patient. Increased patient awareness and education have also led to the formation of various patient registries and services—ResearchMatch,^30^ SmartPatients,^31^ Antidote.Me,^32^ etc—which drive enrollment for specific trials, referred to as patient-driven enrollment. Institution-based enrollment is broad in scope and involves finding appropriate patients for its entire clinical trial portfolio. Alternatively, clinical trial–based enrollment is narrow in scope and usually directed by trial sponsors—that is, pharmaceutical companies, trial investigators, commercial vendors, etc—for a specific trial. All these workflow models require active initiation and maintenance efforts and could be limited by human resources. Setting up automated process triggers to perform a reflex trial matching can kickstart the matching workflow without requiring human intervention. A variety of events can be designated to act as potential triggers (Fig 2). In an ongoing study at Vanderbilt-Ingram Cancer Center, an NGS test result is being used as a triggering event for clinical trial matching. Such built-in processes reduce clinician burden, minimize missed trial opportunities for patients, and thus provide a boost to clinical trial enrollment efforts.

### Clinical Trial Search Tools and Services

As 66% of patients become aware of a clinical trial opportunity through their provider/clinical research staff, it is imperative that clinical trial search tools are available to providers.^33^ There are several private and public databases (Table 1) that support user profiles, send customized study alerts, and allow multifaceted filtering to refine trial results.

Identifying and evaluating the most significant eligibility criteria for a specific disease group and using these to refine trial search results could be an effective strategy for improving trial matching—for example, estrogen receptor/progesterone receptor/human epidermal growth factor receptor 2 status for breast cancer and microsatellite instability status for colorectal cancer. In an internal prescreening study that included 6,277 patients, we found that prior therapies (19%) and treatment setting—that is, neoadjuvant, adjuvant, and metastatic (12%)—were the most likely reasons for false positives in trial matching events (unpublished data). In a private annotation of the Vanderbilt trials, we have manually added treatment context and drugs to all institutional trials to refine matching outcomes.

To receive the full benefit of trial-matching services it is important to integrate them with the EHR. Lack of EHR integration can lead to disruptive workflows and erroneous trial matching as a result of outdated patient values. We envision a workflow in which structured data files from a patient’s NGS tests are directly uploaded into a clinical decision support system that is integrated with the EHR. The system then performs reflex trial matching and suggests clinical trials that are a potential match for the patient.

### Full Match, Partial Match, and Future Match

When running the trial match algorithm for a patient there could be several trials that partially match patient criteria but need additional criteria verification to be a full match. This information may be unknown to the system or may not have been evaluated (eg, a biomarker that was not present on the panel that was used for testing). Combining these partially matched trials can enable an algorithm to create a list of missing biomarkers and rank them on the basis of the frequency of occurrence in trial eligibility criteria. Building matching algorithms that support the ability to partially match to trials can be extremely powerful in informing the decision to perform additional tests and guide the selection of a gene panel to cover the highest number of genes on a patient’s missing biomarker list.

A trial matching event for a patient might also identify clinical trials that may be suitable as the next line of therapy on the basis of treatment setting, disease status, or prior treatments (eg, a second-line metastatic trial for a patient currently receiving first-line therapy). It is helpful for a provider to know about these future match trials so that decisions regarding the current treatment plan do not disqualify the patient from future line trial options.

### Waitlist Management

Waitlist workflows are an important part of the trial recruitment process and are used in multiple scenarios. Common scenarios include tracking patients who are eligible for a trial for which there is no slot currently available, or tracking patients who may be eligible for a trial upon progression of their disease. Current waitlist workflows rely heavily on manually maintained spreadsheets. It is not uncommon for waitlists to contain deceased patients and outdated trial cohort statuses.

An ideal waitlist management system should be integrated with the EHR and CTM, allowing for dynamic updates of cohort level recruitment status for trials and updated patient variables. A nightly refresh should remove deceased patients and closed trial cohorts and allow users to reallocate patients on the basis of the updates. Such systems can dramatically reduce screening efforts when competitive spots open in phase I studies, for example.

### Evaluation Strategies

It is crucial to design appropriate evaluation strategies to assess incremental benefits from adding workflow enhancements to the clinical trial enrollment process. Feedback surveys have been widely adopted to assess benefit. A more rigorous approach using pragmatic study designs could be used to continuously and iteratively evaluate enhancements to tools that support clinical trial matching workflows. TrialProspector used pilot deployment of their tool to a small group of physicians to test usability and performance.^5^ We are currently using a pragmatic randomized study to assess the benefit of providing clinical decision support to oncology providers in terms of possible clinical trial matches for their patients.

## SUMMARY

In this review, we summarize the current state of the clinical trial enrollment workflow and offer our vision of the tools and services that can fill existing gaps. Informatics tools to support the entire clinical trial enrollment workflow—beyond just finding trial matches—are urgently needed to improve efficiency and clinical trial accrual rates. Employing a three-pronged approach of standardizing patient data models, structuring clinical trial documents, and creating resources to access real-time cohort level recruiting status could dramatically improve clinical trial outcomes. Continuous evaluation of the performance of automated clinical trial matching algorithms and iterative refinements will be essential as these tools may perform differently at different institutions.
